# Nuclear Import of Adeno-Associated Viruses Imaged by High-Speed Single-Molecule Microscopy

**DOI:** 10.3390/v13020167

**Published:** 2021-01-22

**Authors:** Samuel L. Junod, Jason Saredy, Weidong Yang

**Affiliations:** Department of Biology, Temple University, 1900 N. 12th Street, Philadelphia, PA 19122, USA; sam.junod@temple.edu (S.L.J.); tug39776@temple.edu (J.S.)

**Keywords:** nucleocytoplasmic transport, super-resolution microscopy, virus-host Interactions

## Abstract

Understanding the detailed nuclear import kinetics of adeno-associated virus (AAV) through the nuclear pore complex (NPC) is essential for the application of AAV capsids as a nuclear delivery instrument as well as a target for drug development. However, a comprehensive understanding of AAV transport through the sub-micrometer NPCs in live cells calls for new techniques that can conquer the limitations of conventional fluorescence microscopy and electron microscopy. With recent technical advances in single-molecule fluorescence microscopy, we are now able to image the entire nuclear import process of AAV particles and also quantify the transport dynamics of viral particles through the NPCs in live human cells. In this review, we initially evaluate the necessity of single-molecule live-cell microscopy in the study of nuclear import for AAV particles. Then, we detail the application of high-speed single-point edge-excitation sub-diffraction (SPEED) microscopy in tracking the entire process of nuclear import for AAV particles. Finally, we summarize the major findings for AAV nuclear import by using SPEED microscopy.

## 1. Introduction

### 1.1. Overview of the NPC and Nuclear Transport of Viruses

Viruses are obligate intracellular pathogens that hijack the cellular components of an infected host cell to produce viral progeny. Production of viral progeny may occur exclusively in the cytoplasm or may incorporate the host nucleus for viral replication. Typically, this would involve viruses delivering their genetic materials, including DNA or RNA, into the nucleus through the nuclear pore complex (NPC) [1], a complex protein structure embedded in the nuclear envelope (NE). The NEs of mammalian cells contain thousands of NPCs [2,3], which serve as the major pathway for transiting molecules to cross the NE [4,5]. The NPC consists of ~30 different types of proteins, known as nucleoporins (Nups), possessing a total molecular weight of ~60–120 MDa [6]. The eight-fold stoichiometry of the scaffold Nups [7] gives the NPC an hourglass shape with a diameter of ~50 nm within the narrowest central region [8,9,10,11]. Anchored to the scaffold Nups are intrinsically disordered Nups with varying concentrations of repeating phenylalanine–glycine (FG) residues [8]. Based on the primary core sequence of different FG repeats, FG Nups can be divided into subfamilies or subtypes. The major types include FxFG (phenylalanine-x-phenylalanine-glycine), GLFG (glycine-leucine-phenylalanineglycine), and xxFG (x-x-phenylalanine-glycine) [2,8]. Spatially, these FG-Nups are assembled in three distinct locations in the NPCs. In vertebrate cells, nuclear basket FG-Nups, Nup153, Nup50, and Tpr occupying the nucleoplasm side of the NPC contain only FxFG motifs. Cytoplasmic FG-Nups, hCG1, Nup358, and Nup214 containing both FxFG and xxFG motifs situate towards the cytoplasmic side of the NPC. Lastly, central FG-Nups, Nup98, POM121, Nup58/45, Nup54, and Nup62 possessing the xxFG and GLFG motifs are located towards the center of the NPC [2,8,12].

These dynamic FG-Nups create a hydrophobic barrier within the NPC that prevents large molecules (>40–60 kDa) from passively diffusing through the NPC [13]. In order to conquer this barrier, large molecules must contain either a nuclear localization signal (NLS) for nuclear import or a nuclear export signal (NES) for nuclear export [14]. Transport receptors may recognize these signals and ferry the cargo through the NPC by direct interactions with the FG-Nups. Following nuclear import of a cargo-receptor complex, RanGTP will facilitate cargo-receptor disassembly through an allosteric mechanism. Alternatively, RanGDP by GTP hydrolysis will disassemble the cargo-receptor complex following nuclear export. The concentrated RanGTP and RanGDP—respectively in the nucleus and the cytoplasm—function as the nuclear transport direction regulators. In contrast, small molecules (<~40 kDa) will passively diffuse through the NPC with neither the help from transport receptor nor the consumption of energy [13].

**Figure 1:**
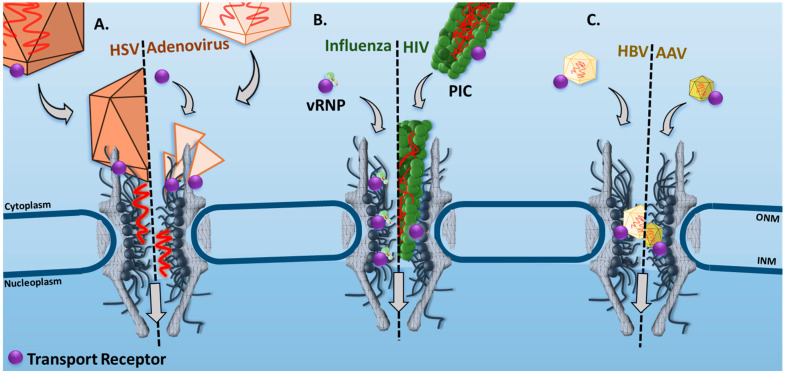
Involvement of transport receptors in viral genome translocation through the nuclear pore complex (NPC). (**A**) Herpes simplex virus (HSV) and adenovirus capsid dock to an NPC through interacting with cytoplasmic nucleoporins (Nups) and then inject viral genome into the nucleus. (**B**) Facilitated transport of influenza’s viral ribonucleoprotein (vRNP) and human immunodeficiency virus’s (HIV’s) pre-integration complex (PIC) through the NPC. (**C**) Facilitated transport of hepatitis B virus (HBV) core protein and adeno-associated virus (AAV) capsid into the nucleus. The viral particles—HSV-1 [15], adenovirus 2 [20], Influenza A vRNP [22], HIV-1 PIC [23], HBV viral core [24], and AAV2 [25]—have been scaled to the NPC dimensions [9]. The transport receptors and the distance between NPCs are not to scale. ONM and INM indicate the outer nuclear membrane and inner nuclear membrane, respectively.

Although the nuclear translocation mechanisms for a viral genome to be delivered into the nucleus may vary between viral classes, transport receptors have been associated with the docking at the NPC and/or facilitated translocation through the NPC. In the case of herpes simplex virus 1 (HSV-1), the capsid (~125 nm in diameter [15]) contains an NLS on the viral protein VP1–2 that is firstly recognized by importin beta 1 (Impβ1) [16]. Then, the HSV capsid will dock to an NPC through the interactions between cytoplasmic Nup358 and Impβ1 [17,18]. Subsequently, cytoplasmic Nup214 will interact with the capsid as well, and the viral genome will be injected into the nucleus, verified by an RNAi knockdown of Nup214 resulting in low translocation of viral DNA (Figure 1A) [19]. A similar process has been shown for the adenovirus 2 capsids (~95 nm in diameter [20]), in which the adenovirus 2 capsid will disassemble and recruit transport receptors, such as the Impα and Impβ1 complex, to interact with cytoplasmic Nup214 and translocate the viral genome into the nucleus (Figure 1A) [21].

In the case of human immunodeficiency virus 1 (HIV-1), its nuclear translocation is accomplished by the formation of a pre-integration complex (PIC, conical shape ~120 × 60 × 40 nm in three dimensions [23]) with an NLS-tagged matrix protein and virion-associated accessory protein, VpR [26,27]. This suggests recruitment of transport receptors for facilitated transport through the NPC [28,29,30] (Figure 1B). Similarly, influenza A will form the viral ribonucleoprotein (vRNP, ~10–15 nm in diameter [22]) complex, consisting of two NLSs that will be recognized by Impα and Impβ1 transport receptors [31,32] (Figure 1B). 

Finally, some parvoviruses, such as adeno-associated virus 2 (AAV2, ~25 nm in diameter [25]) (Figure 1C), will hijack the facilitated transport mechanism for the translocation of the entire viral capsid into the nucleus [33]. This mechanism is likely explained by possible NLSs in the VP1, VP2, and VP3 basic regions, which, when mutated, significantly reduce import of the viral capsid [34]. Hepatitis B virus (HBV) core (~36 nm in diameter [24]) may transport by a similar mechanism via a C-terminal NLS on the core proteins of the virus that is exposed during phosphorylation (Figure 1C) [35,36]. It is noteworthy that there are also instances in which parvoviruses have been shown to disrupt the NE by caspases, bypassing the NPC, to gain access to the nucleus [37,38].

### 1.2. Live Cell Imaging with High Spatial Resolution Is Needed to Detail Viral Nuclear Transport Kinetics through the NPC

To use viral vectors as a nuclear delivery mechanism, it is critical to understand the kinetics of viral import through the NPC into the nucleus. Molecular dynamics in live cells have been well studied by using conventional fluorescence microscopy, in which molecules of interest are typically tagged with fluorescent proteins or dyes. However, due to the Abbe diffraction limit, the spatial resolution of light microscopy is approximately half of the wavelength of the emission light. If a 500 nm emission light is collected, the spatial resolution of ~250 nm in the x and y dimensions and about ~750 nm in the z dimension will be expected [13,39,40,41,42,43,44]. Such limited spatial resolutions of conventional light microscopy will prevent distinguishing individual macromolecules moving through the sub-micrometer NPC in live cells [45]. In contrast, electron microscopy (EM) can provide a high spatial resolution of up to <1 nm; however, chemical fixation or freezing of samples makes it almost impossible to achieve the real-time dynamics in live cells [46,47,48]. Thus, new techniques are urgently needed to break these limitations and further study the in vivo interactions between the viral particles and the nuclear pores.

### 1.3. Overview of Fluorescence Microscopy Techniques Applied to Study Viral–NPC Interactions

As shown in Table 1, by using different fluorescent microscopy methods, several research groups have been able to reveal new information regarding viral–NPC interactions. In the studies of the interactions between HSV-1 and Nups, D. Pasdeloup et al. employed confocal laser scanning microscopy (CLSM) in combination with fluorescently labeled capsid proteins [19]. With RNAi of several cytoplasmic, central, and nuclear Nups, they found that capsid protein pUL25 interactions with cytoplasmic Nup214 were essential for effective HSV-1 viral genome nuclear import. CLSM was also used by B. Rabe et al. to study the nuclear import of intact HBV capsids [49]. To fully understand the components of the HBV, the authors used six different forms of the viral capsids. They found that import of the virus was dependent on the phosphorylation of the capsid proteins and the recruitment of Impβ1 and Impα. Additionally, they found the capsid to interact with nuclear basket Nups and that it was independent of the GTP-binding protein, Ran.

Fluorescence resonance energy transfer (FRET) is a distance-dependent physical process for probing an inter- or intra-molecular distance of <10 nm by measuring the non-radiative energy transfer between a pair of donor–acceptor fluorophores. By using the FRET technique, M. Martin-Fernandez et al. revealed sub-nanometer changes in localization dynamics of the adenovirus 5 capsid proteins [50]. In the study, they found that adenovirus 5 capsid has two disassociation steps, suggesting a two-step capsid disassembly before viral genome nuclear import. 

To study the nuclear transport kinetics of Influenza A vRNPs at the single-molecule level, single vRNPs were tracked as they interacted with the NE or the NPC in live cells. These studies revealed dissociation rate constants ranging from 0.01 to 1 s^−1^ for binding between the vRNPs and the NE. In addition, a significant reduction in vRNP nuclear localization was observed in the presence of anti-NPC or wheat germ agglutinin (WGA), suggesting the NPC to be the primary transport route of vRNPs. Both anti-NPC [49] and WGA [54] inhibit nuclear transport by physically blocking the central channel of the NPC. For anti-NPC, this is accomplished by protein–protein interactions with central-channel Nups. WGA, a lectin, will bind to O-GlcNAc (O-linked β-N-acetylglucosamine) sites that have been post-translationally added to Nups [55]. 

Moreover, to observe the nucleocapsid interaction of HIV-1 with the NPC and subsequent nuclear import of the PIC, A. C. Francis and G. B. Melikyan utilized stimulated emission depletion (STED) microscopy, one of the patterned-optics-based super-resolution techniques [52]. STED microscopy introduces a doughnut-shaped, STED laser beam to significantly reduce the size of the effective point spread function (PSF), resulting in super-resolution localizations. By observing the co-localization of fluorescently labeled integrase-sfGFP, capsid protein-DsRed, and single HIV-1 viral particles, the authors concluded that the HIV-1 capsid will dock to an NPC, shed capsid proteins, and import the PIC into the nucleus.

## 2. Description of Methods

### SPEED Microscopy Illuminates Individual AAV Molecule Transport through Single NPCs

The studies mentioned in Section 1.3 have provided great insights into the dynamics of viral import by imaging single particles across the NE in live cells. However, the AAV vectors continue to be the best nuclear gene delivery instrument for the treatment of human diseases due to their low pathogenicity and tissue specificity [56]. This motivated our group to refine the imaging process of AAV nuclear import by investigating more details inside the sub-micrometer NPCs. These details included the nuclear import time, the nuclear import success rate, and the configuration of AAV particles through single NPCs in live cells [33]. To obtain these kinetics, our lab has developed single-point edge-excitation sub-diffraction (SPEED) microscopy and applied it to conduct single-particle tracking (SPT) of translocation of single AAV molecules through single NPCs in live HeLa cells. SPEED microscopy involves new technical developments in both microscopy imaging and single-molecule data analyses [13,57].

**Figure 2:**
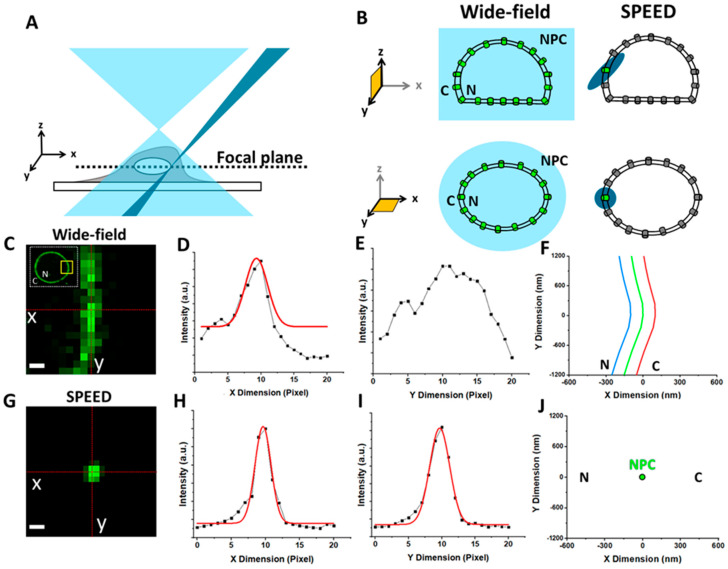
Illumination patterns of wide-field and single-point edge-excitation sub-diffraction (SPEED) microscopy techniques. (**A**) Diagramed illumination volumes of wide-field (light blue) and SPEED (blue) microscopy. (**B**) GFP-labeled NPCs embedded in the NE were highlighted inside (green) and outside (gray) the microscopy illumination volumes in the xy and yz planes [60]. (**C**) Selected area (yellow box) of GFP–NE florescence from wide-field illumination. The x- and y- axes are shown as dashed red lines intersecting at the NE equator. Scale bar, 0.5 μm. (**D**,**E**) Intensities from a line scan of pixels (dashed red lines) in the x dimension and y dimension. (**F**) The middle plane of the NE, determined by the peak x dimension and y dimension intensities, is shown as a green curve. The blue and red curves refer to −100 and +100 nm from the middle plane of the NE, respectively. (**G**) Single GFP–NPC excited with the illumination volume of SPEED microscopy. (**H**,**I**) The peak x and y position of the 2D line-scanning fitting from a single GFP–NPC fluorescent spot. (**J**) The 2D centroid of a single NPC determined by 2D Gaussian fitting. **N**, nucleus; **C**, cytoplasm. Figures were adapted from our previous publications with permission [61].

The average distance between human NPCs is around 400–600 nm and limits the use of epifluorescence microscopy due to indistinguishable overlapped fluorescence from neighboring fluorescence-protein-labeled NPCs [58,59]. Because of this overlap, detailed translocation kinetics for AAV particles moving through single NPCs are difficult to resolve. To overcome this limitation, an inclined diffraction-limit illumination point spread function (iPSF) of SPEED microscopy was generated, which allows for the illumination of a single NPC by the iPSF in three dimensions. Technical advances in SPEED microscopy are further detailed as follows [33,57]: (1) Compared to wide-field microscopy, SPEED microscopy generated a diffraction-limited iPSF (~320–230 nm in the x, y, and z directions when using a tilted laser with an angle between 35° and 55° for a 488 nm excitation laser) that is smaller than the average distance between NPCs on the NE (Figure 2A,B). (2) Because of the small inclination of the iPSF of SPEED microscopy, a very fast detection speed (up to 0.2 milliseconds per frame) was achieved by using a small pixel area of a charge-coupled device (CCD) camera to track single molecules going through single NPCs. (3) The high laser power density (100–500 kW/cm^2^) at the focal plane also produced a high number of photons within a short detection time from a single AAV2 particle labeled with approximately 36 Alexa Fluor 647 dyes. With the high laser power density and label efficiency, more than 5000 photons were collected from a single AAV2 particle within a 2 ms detection time. (4) The inclined iPSF greatly avoided out-of-focus background fluorescence and auto-fluorescence of the objective, resulting in a significantly improved signal-to-noise (SNR) ratio [60]. (5) Photobleaching and phototoxic effects in live samples were greatly reduced by the unique pinpointed illumination pattern of SPEED and an on/off operational mode of the excitation laser with an off-time that is ten-fold longer than the on-time. Altogether, these features enabled SPEED microscopy to image AAV particles in live cells with a spatiotemporal resolution of 2 ms and <10 nm.

## 3. Discussion

### 3.1. New Features for the Nuclear Transport of AAV Particles Obtained by SPEED Microscopy

**Figure 3:**
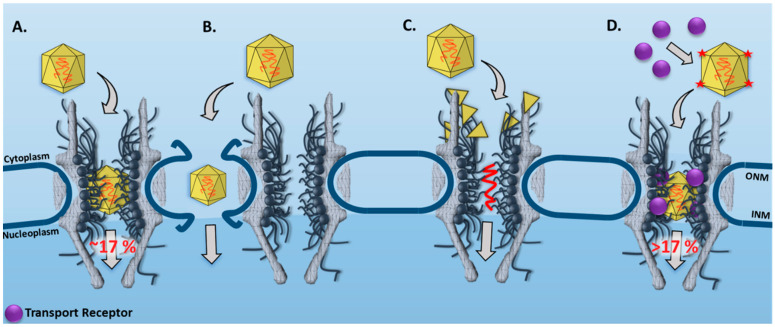
New features for the nuclear transport of AAV obtained by SPEED microscopy. (**A**) Our measurements revealed that approximately 17% of AAV2 capsids can be intact and successfully imported through the NPC to reach the nucleus. (**B**) A model in which AAV2 might bypass the NPC and gain access to the nucleus by disrupting the NE. (**C**) Another model in which the AAV2 capsid could disassemble on the cytoplasmic side of the NPC and inject viral genome into the nucleus through the NPC. (**D**) A suggested approach to increase the nuclear import success rate for AAV2 capsid as a nuclear gene delivery instrument. The transport receptors, the distance between NPCs, and the AAV2 particles are not to scale. ONM and INM indicate the outer nuclear membrane and inner nuclear membrane, respectively.

In our study, we found significant evidence for nuclear import of intact AAV2 capsids by tracking individual AAV2 particles across the NE and moving through single NPCs in live cells (Figure 3A) [33]. Our data revealed that approximately 17% of the intact AAV2 particles starting from the cytoplasm successfully transverse the NPC to reach the nucleoplasm (Figure 3A). Compared to the higher transport efficiency of transport receptor Impβ1, ~50% [13], we concluded that the nuclear import of AAV2 may be another rate-limiting step for AAV2 transduction. The other steps, including the cytoplasmic membrane [62] and the endosomal membrane [63], have been suggested as potential barriers to limit AAV2 transduction rates.

Throughout our experiments, we neither witnessed membrane invaginations on the NE by AAV2 particles nor their disassembly on the cytoplasmic side of the NPC (Figure 3B,C). By observing the GFP-labeled NE with a wide-field illumination area, we found the fluorescently labeled NE remained continuous during AAV2 interaction with the NE. If the Alexa Flour 647 labeled AAV2 capsid were to disrupt the NE, we would have observed an absence of GFP fluorescence at the Alexa Flour 647 labeled AAV2 particle. With a smaller illumination area, we confirmed the capsid’s completeness by observing a constant fluorescence intensity profile consistent with a singular moving fluorescently labeled particle, suggesting that the AAV2 capsid remained intact during nuclear import. 

Achieving a higher nuclear import success rate for the AAV2 capsid through the NPC would be a critical step for AAV2 to function as a nuclear gene delivery instrument. Previously, several studies have suggested that additions of different quantities and/or different types of NLSs on large cargo particles could improve their nuclear import efficiencies through the NPC [64,65]. Since intact AAV2 capsids were found to transport into the nucleus [33], engineering AAV capsid proteins with more and different types of NLSs may have a positive effect on AAV nuclear import (Figure 3D). Additionally, recently, we found that the nucleocytoplasmic transport of intrinsically disordered proteins (IDPs) was independent of molecular sizes and transport receptors that are well executed for folded proteins. Instead, the number of the charge and hydrophobic amino acid residues of the IDPs dominates their nuclear transport mechanisms [66]. In detail, the IDPs with a higher concentration of hydrophobic residues diffused through the NPCs with higher successful transport rates. Thus, another alternative approach for improving the nuclear import efficiency of AAV2 capsids could be increasing the external hydrophobicity of the AAV2 capsids.

### 3.2. Limitations of SPEED Microscopy 

SPEED microscopy has answered critical biological questions surrounding the NPC; however, there are still a few limitations in the operation and application of the method. The first limitation is the alignment of multiple lasers to form an inclined illumination pattern in the focal plane of the optical objective. This requires a good understanding of optics and microscopy for the placement of multiple reflection mirrors, dichroic filters, and optical beam steering. Another limitation is the large amount of time needed for collecting/analyzing data and validating experimental results through computational simulations. Typically, for determination of fast dynamics in live cells, hundreds of single-molecule trajectories are needed for reproducible transport times and efficiencies. To obtain sub-micrometer subcellular structural information, thousands of single-molecule localizations with high localization precisions need to be collected and analyzed. After data collection, post-localization transform algorithms or/and simulations will be applied to obtain super-resolution structural and dynamic information. The complete protocol includes cell culture and sample preparation, SPEED microscopy imaging, data analysis, and validation through simulation, which takes approximately nine days to complete [67].

## 4. Description of the Equipment 

### 4.1. Microscopy

An Olympus IX81 microscope was used to perform our experiments. However, any high-end inverted microscope with a camera port for a CCD camera, epi-fluorescence port for wide-field imaging and scanning, and a side port for excitation lasers may be used as well. Our microscope was equipped with 1.4 numerical aperture (NA) 100x oil-immersion apochromatic objective (UPLSAPO 100XO; Olympus). A high NA objective is not necessary for single-molecule microscopy, but will significantly improve localization precision. The stage clips (IX-SCL) were used to clamp the samples on an Olympus IX-SVL2 stage. A well-calibrated stage is necessary for single-molecule experiments; otherwise, the sample may shift away from the focal plane during imaging. Optical axis plane movement was controlled by a focus adjustment knob. Sample plane (x and y) movement was controlled by a mechanical IX-SVL2 stage. A 100 W halogen lamp was used for bright-field illumination. A 100 W mercury lamp was mounted to the epifluorescence excitation port. The entire SPEED microscope system was isolated from vibrations by mounting on a pneumatic isolator that was pre-mounted on a research-grade optical table (Newport, Irvine, CA, USA). The microscope was mounted on a secondary passive vibration control (Herzan; Onyx Series) to dampen vibrations created by equipment on the optical table. 

### 4.2. Laser Illumination and Filter Setting

Two lasers were used in the above microscope system to provide the power necessary for rapid image acquisition of single-molecule fluorescence signals. Lasers included a 35 mW 633 nm HeNe laser (Melles Griot) and a 50 mW 488 nm continuous-wave solid-state laser (Coherent; OBIS Series). Linearly polarized laser light was converted into circularly polarized light with a quarter-wave plate to allow homogeneous (polarization-independent) fluorophore excitation within the focal plane. After converting into a circularly polarized light, laser output was modulated with a neutral density filter. Laser power should be measured after the last neutral density filter for an accurate reading for imaging experiments. After the neutral density filter, an optical chopper (Newport) was used to generate an on–off mode of laser excitation. To minimize alignment problems in dual-color measurements, both green and red fluorescence emissions were collected by the same objective and filtered by a dichroic filter (Di01-R405/488/561/635–25 × 36; Semrock) and an emission filter (NF01–405/488/561/635–25 × 5.0; Semrock).

### 4.3. CCD Camera

In our experimental setup [33], an on-chip electron-multiplying charged-coupled device (EMCCD) camera was used for signal detection. CCDs provide wide-field spatial information that cannot be obtained with photomultiplier tubes or avalanche photodiodes. Frame rates are largely limited by the frame-transfer speed and pixel number of a camera. We chose a 128 pixel × 128 pixel CCD camera (Cascade128+; Roper Scientific) to track moving AAV2s. For this camera, full-frame and continuous image acquisition occurs at 500 frames per second (fps), with >90% quantum efficiency. Faster frame rates can be obtained by limiting the acquisition area using software: e.g., for a 128 pixel × 20 pixel area, 2500 fps can be acquired. Dark current is a major factor limiting image quality for EMCCDs, especially at high frame rates. A solution is used to cool down the detection chip. The Cascade128+ camera is cooled to −30 °C, leading to a dark current of ≤1 e^-/p/s^. 

### 4.4. Imaging Software

For our image acquisition, we used the Slidebook (Intelligent Imaging Innovations) software for data acquisition, instrument control, and most image processing. Typically, image acquisition software is associated with the selected camera. We recommend the software that is compatible with both the image acquisition and the motorized components of the microscope, such as a z-stepper, objective changer, filter changer, and emission path changer (e.g., eye to camera or camera to camera). Additional instrumentation add-ons that we recommend include a laser shutter and motorized stage. The laser shutter, properly integrated into the imaging software, can reduce unintentional photobleaching between videos by blocking the exciting laser beam. The motorized stage can monitor cell drift caused by fluctuations in the optical setup and simplifies laser alignment. We used Glimpse, a MatLab-based program written by Jeff Gelles, to fit fluorescent emission spots with two-dimensional Gaussian distributions for particle tracking purposes [33,68]. Separately, we also used the open-source GDSC single-molecule software found in ImageJ Fiji [69]. The GDSC software may be used for both single-molecule localizations and trajectories, and is described further in Section 5.6 and Section 5.8, respectively. 

## 5. Experimental Procedures

### 5.1. Preparation of AAV Particles

AAV was originally discovered in 1965 by Hammon [70] and in 1966 by Rowe [71] as a potential subunit contaminant of adenovirus preparations. The reader is directed to an excellent overview of the early history and use of AAV as a viral vector by Barrie Carter [72]. Due to the nature of AAV being non-pathogenic and replication defective, the virus is an excellent choice for building viral vectors requiring less than 4.7 kb packaging space. As it is replication defective, the virus requires additional helper proteins that are expressed by helper viruses, such as adenovirus or herpes virus. 

#### 5.1.1. Production of AAV Particles

Production of AAV virions can be performed either with a helper virus or plasmids containing the required helper proteins. The former introduces potential immunogenicity issues if downstream applications are for in vivo use, and also reduces biosafety concerns with a functional helper virus during rAAV packaging [73]. The helper-free protocol requires the use of three vectors: (1) promotor and gene of interest (GOI) flanked by two AAV ITR sequences denoted as pAAV-GOI, (2) a helper plasmid expressing adenovirus E2A, E4, and VA genes denoted as pHelper, and (3) AAV Rep and Cap gene denoted as pRC (note: Cap has various native and modified serotypes for targeting specific cell types). In our single-virus imaging protocol, we introduced a fourth plasmid containing an AVI peptide sequence inserted into pRC and *E. coli* biotin holoenzyme synthetase (BirA). Packaging rAAV using a helper-free protocol must be done in a cell line stably expressing Adenovirus E1 protein, of which there are several derivations of HEK293. The protocol here lists using HEK293A (ThermoFisher Cat #R70507, Thermo Fisher Scientific, Waltham, MA, USA) and volumes for a T-150 flask with polyethylenimine (PEI) transfection. The reader may need to adjust seeding density according to their desired transfection reagent and flask size. Usage of AAV as a recombinant vector (rAAV) removes both AAV proteins Rep and Cap, making rAAV nonlytic; thus, incubation post-transfection is typically 48–120 h.

1. Prepare purified plasmids pAAV-GOI, pRC, pHelper, and pBirA-EGFP. The pRC has the AVI peptide sequence inserted into AA139–140 of the basic region (BR1) location.
2. Culture HEK293A cells in DMEM high glucose with L-glutamine and 1 mM pyruvate (Gibco), supplemented by 10% (*v/v*) Fetal Calf Serum (FBS) and 1% (*v/v*) Antibiotic–Antimycotic. Plate and incubate HEK293A cells at 37 °C within a 5% CO_2_ incubator until 70–80% confluent.
3. With calcium phosphate transfection, insert pAAV-GOI, pRC, pHelper, and pBirA-EGFP into the HEK293A cells. First, mix a 1:1:1:2 molar ratio of pAAV-GOI:pRC:pHelper:pBirA-EGFP for a total DNA concentration of 25 µg. Adjust the total volume of DNA to 25 µL.
4. Mix 30 µL of CaCl_2_ (2.5 M), 245 µL H_2_O, and 25 µL of the DNA. Add the DNA mixture dropwise to bubbling 300 µL of 2X HEPES-buffered saline (HBS; 50 mM HEPES [*N*-2-hydroxyethylpiperazine-*N*′-2-ethanesulfonic acid], 280 mM NaCl, and 1.5 mM Na_2_HPO_4_, pH 7.05) [74]. To bubble the HEPES-buffered saline, use a Pasteur pipette to slowly inject air into the solution as the DNA mixture is added.
5. Vortex the mixture and allow it to sit for 20 min. Confirm that the precipitate is fine with little aggregation. If aggregation does occur, adjust the pH of 2X HEPES to produce a fine precipitate.
6. Add this mixture dropwise to the HEK293A cells and incubate the cells at 37 °C within a 5% CO_2_ incubator for at least 16 h undisturbed.
7. Replace the cell culture media and incubate for an additional 24–48 h.
8. rAAV is nonlytic, so the media will have low titer of virus. After incubation, gently knock the plate to lift the cells. Alternatively, add EDTA (0.5 M) to help lift the cells. If a higher titer is desired, retain cell media and add to the AAV precipitation step.
9. To ensure effective labeling of biotin on AAV2 capsid proteins, fluorescence-activated cell sorting (FACS) is used to separate the transfected HEK293A cells expressing EGFP from the other cells.
10. Centrifuge the fluorescently labeled cells at 500× *g* for 5 min. Then, resuspend in 1 mL of phosphate-buffered saline (PBS; pH 7.4).
11. Lyse the cells using a freeze/thaw method of 10 min in liquid nitrogen or a dry ice–ethanol bath, then transfer to a 37 °C water bath until the cellular mixture is completely thawed. Repeat the freeze/thaw three more times (HINT 1).
12. After the last thaw at 37 °C, add 50 U/mL benzonase and 10 U/mL RNase I to the virus-released solution. Incubate for 30 min at 37 °C in a water bath. Then, add 0.5% sodium deoxycholate and incubate for an additional 30 min.
13. Centrifuge the mixture at 10,000× *g* for 10 min and save the supernatant. Add PEG 8000 and NaCl for a final concentration of 8% PEG 8000 and 0.5 M NaCl. Then, incubate on ice for 60 min.
14. Centrifuge AAV particles at 2000× *g* for 30 min at 4 °C. Then, resuspend the pellet in a low volume of HBS (pH 8.0). The crude pellet can be stored at −80 °C.

#### 5.1.2. Purification of AAV Particles

Purification of rAAV can be accomplished by either ultracentrifugation methods [75,76], for which the reader is directed to the referenced material, or the two-phase separation method described here.

1. Add an equal volume of chloroform to the crude rAAV pellet. Vortex vigorously for 2 min until a homogenous mixture is obtained. Then, centrifuge for 5 min at 370× *g* and retain the aqueous phase. In a sterile environment, vent the aqueous phase for 30 min. This will evaporate the remaining chloroform.
2. Dilute aqueous phase into 10%PEG8000/13.2% (NH_4_)_2_SO_4_ ((*w/w*), pH 8.0) (HINT 2).
3. Let the mixture incubate for 15–30 min at room temperature, then centrifuge at 3000× *g* for 15 min. Then, carefully draw out the clear bottom phase with a needle. The bottom clear phase is the virus-containing phase.
4. Concentrate this phase with centrifugal filters (Amicon Ultra-0.5 mL Centrifugal Filters; Ultra 50K device). At room temperature, centrifuge the sample for 20 min at 14,000× *g*. Rinse the sample twice with a low-salt solution (NaCl (10 mM), pH 7.4).
5. Store the final concentrated rAAV stock at −20 °C in PBS (pH 7.4) or minimum essential media (MEM; Gibco, Thermo Fisher Scientific, Waltham, MA, USA) with 0.001% (*v/v*) pluronic F68 (Gibco).

#### 5.1.3. Labeling of AAV Particles 

By using the AVI peptide sequence insert on 139–140 in the pRC plasmid with the BriA-EGFP plasmid, we were able to label the AAV2 capsid with biotin. The co-transfection of a AVI tag plasmid with a BriA plasmid will produce biotin sites at the AVI sequence inserts by BirA enzymatic biotinylation of a lysine side chain of the AVI tag [77,78]. The FACS of the HEK293A will isolate most of the cells expressing the BirA-EGFP protein, so the final purified AAV2 particles should be biotin-labeled and can be labeled with the Alexa Fluor 647 streptavidin through a biotin–streptavidin interaction.

1. At a concentration ratio of 40:1 (streptavidin–biotin), incubate the Alexa Fluor 647-labeled streptavidin with biotin-labeled AAV2 particles at 4 °C while agitating for 1 h while covered from light.
2. Isolate the labeled AAV2 particles from free streptavidin by centrifugal filtration (Amicon Ultra-0.5 mL Centrifugal Filters). We recommend the Amicon Ultra 50K device.
3. At room temperature, centrifuge the sample for 20 min at 14,000× *g*. Rinse the sample twice with PBS (pH 7.4). Then, measure the labeling efficiency of the AAV2 particles, as shown in Section 5.5.

### 5.2. Preparation of Purified Proteins

To investigate the linkage between the transport receptor, Impβ1, and AAV nuclear import, we purified Impβ1 through a 6X-Histidine-tagged protein and gravity-flow chromatography with Ni-NTA Superflow (Qiagen, Hilden, Germany). As stated in the introduction, several viruses importing into the nucleus have been associated with transport receptors. Purification of the transport receptors is a necessary step to investigate the connection between viral particles and individual transport receptor types. 

#### 5.2.1. Bacterial Transformation and Expression

The Impβ1 plasmid contains a T7 promoter region, which can be activated by Isopropyl-β-Δ-thiogalactopyranoside (IPTG), a N-terminal 6X-Histidine tag, and the ampicillin-resistance sequence.

1. Transform BL21 (DE3) (New England BioLabs Inc., Ipswich, MA, USA) *E. coli* cells with ~0.1–100 ng of Impβ1 plasmid DNA through a heat shock at 42 °C for 10 s.
2. Incubate the transformed BL21 (DE3) cells for 60 min at 37 °C in a 19:1 dilution of Super Optimal Broth (SOC, 0.5% Yeast Extract, 2% Tryptone, 10 mM NaCl, 2.5 mM KCl, 10 mM MgCl_2_, 10 mM MgSO_4_, 20mM Glucose) Media to transform BL21 (DE3) cells.
3. Spread 50 µL of the mixture onto ampicillin (100 mg/mL) agar plates and incubate at 37 °C overnight (12–14 h) (HINT 3).
4. Create a starter culture from a single bacterial colony in 5 mL of Lysogeny Broth (LB) with the addition of 5 μL of ampicillin (100 mg/mL) and grow aerobically with shaking overnight (12–14 h) at 37 °C at 225 rpm.
5. Transfer the 5 mL saturated starter culture to 1 L of LB media with 1 mL ampicillin (100 mg/mL). Shake the mixture at 37 °C until an OD 600 nm of ~0.6 is reached, usually 5–6 h. Add 1 mL of IPTG (1 M) to activate protein production and incubate overnight at 30 °C (HINT 4).

#### 5.2.2. Protein Purification

Centrifuge the culture at 4000× *g* for 10 min at 4 °C and discard the supernatant. Resuspend the pellet with CelLytic B (Sigma-Aldrich, St. Louis, MO, USA) or Lysis buffer (50 mM NaH_2_PO_4_, 300 mM NaCl, 10 mM Imidazole, pH 8.0) with 10 mL of buffer to 1 g of pellet (HINT 5).

To inhibit protein degradation, add a “protease inhibitor cocktail” (400 μL of 0.2 mg/mL pepstatin A; 400 μL of 0.2 mg/mL leupeptin; 400 μL of 2 mg/mL trypsin inhibitor) to the resuspended cells.

Fractionate the bacterial membrane layer using chemical (B-PER Bacterial Cell Lysis Reagents) or physical fractionation. For physical fractionation via the high-pressure Avestin Emulsiflex B15 (ATA Scientific, Taren Point, NSW, Australia), use a chamber pressure of 50 PSI, cycle the resuspended cells through the system three times, and place the lysate on ice after fractionation. 

Centrifuge the lysate for 10 min at 4 °C and >12,000 g, and discard the pellet. 

Prepare Ni-NTA resin (Qiagen) by removing the ethanol; it is stored via centrifugation at >12,000 g for 1 min followed by aspiration of the supernatant and resuspension with CelLytic B (Sigma-Aldrich, St. Louis, MO, USA) or Lysis buffer. Repeat this process three times. Then, add the Ni-NTA solution to the protein mixture.

Stir the mixture in the dark and on ice for 60 min to allow an adequate time for the binding reaction between the histidine tag and Ni-NTA beads, then centrifuge at 12,000× *g* for 60 min at 4 °C.

Resuspend the pellet in 20 mL of 4 °C cooled Lysis buffer, then transfer to a polypropylene column. Collect the eluate. 

Wash the column twice with 2.5 mL of 4 °C cooled Lysis buffer. Collect the eluate. 

Use an elution buffer (50 mM NaH_2_PO_4_, 300 mM NaCl, 20 mM Imidazole, pH 8.0) with increasing Imidazole concentration of 20 mM for the starting fraction and 250 mM for the final fraction. Collect a minimum of 10 fractions of 0.5 mL eluate. 

#### 5.2.3. Protein Characterization

To characterize the Impβ1 protein, we used both the NanoDrop 2000/2000c (ThermoFisher) UV spectrophotometer and SDS-PAGE. 

Using the NanoDrop 2000/2000c (ThermoFisher) or other UV spectrophotometer, measure the A280 of the flow through fractions, and wash and sample them. For an accurate reading, dilute the sample so the absorbance is below 1.2. 

Prepare an 8% polyacrylamide gel (4.7 mL DI-H_2_O, 2.7 mL Acrylamide/Bis (30% (*w/v*), 37.5:1), 2.5 mL Tris-HCl (1.5 M, pH 8.8), 100 µL SDS (10% (*w/v*)), 10 µL N,N,N′,N′-tetramethylethylene-diamine (TEMED), and 32 µL Ammonium Persulfate (APS, 10% (*w/v*)) for the resolving fraction of the gel. Then, once the gel has mostly polymerized, prepare a 4% polyacrylamide gel (6.1 mL DI-H_2_O, 1.3 mL Acrylamide/Bis (30% (*w/v*), 37.5:1), 2.5 mL Tris-HCl (0.5 M, pH 6.8), 100 µL SDS (10% (*w/v*)), 10 µL N,N,N′,N′-tetramethylethylene-diamine (TEMED), 100 µL Ammonium Persulfate (APS, 10% (*w/v*)). 

Prepare the samples by mixing ≤20 µg of lysate or ≤2 µg of purified sample with 5X Loading buffer (300 mM Tris-HCl pH 6.8, 25% (*w/v*) BME, 10% (*w/v*) SDS, 50% (*w/v*) glycerol) in a 5:1 sample to loading buffer ratio. The total volume is dependent on well depth, but <50% of well volume is preferred to prevent cross-contamination by sample well overflow. 

Before loading the samples, boil the samples for 5 min at a rapid boil. After loading, run the gel at 80–100 V until the progression line leaves the stacking buffer. Then, increase the voltage to 120–200 V until the progression line is 1–2 cm from the bottom of the gel. 

Incubate the gel in staining solution (3 mM Coomassie Brilliant Blue R-250 Dye, 45% (*v/v*) methanol, 10% (*v/v*) glacial acetic acid) for 60 min. Rinse the gel with a wash solution (50% (*v/v*) methanol). Incubate the gel for 60 min, or until the bands are clearly visible, in a destaining solution (40% (*v/v*) methanol, 10% (*v/v*) glacial acetic acid).

#### 5.2.4. Protein Desalting and Storage

Isolate the sample fractions that contain the highest concentration of Impβ1 with the smallest amount of impurities. For single-molecule experiments, a protein purity above 90% and a final concentration of ≥10 µM are needed. To ensure protein stability, the sample fractions need to be concentrated and desalted.

1. To further isolate Impβ1 by centrifugal filters (Amicon Ultra-0.5 mL Centrifugal Filters), select a filter that will collect the target protein in the loading chamber while the contaminants elute through. For Impβ1, we used an Amicon Ultra 50K device, since Impβ1 has a molecular weight of ~97 kDa.
2. At room temperature, centrifuge the sample for 20 min at 14,000× *g*. Rinse the sample twice with a low-salt solution (NaCl (10 mM), pH 7.4).
3. Recover the concentrated target protein by inverting the filter into a clean tube and centrifuge for 2 min at 1000× *g*.
4. Resuspend the protein in one of the following solutions for storage [79]:
  - for 24 h at 4 °C, PBS (pH 7.4);
  - for one week at 4 °C, PBS (pH 7.4) with a bacteriostatic agent (0.1% (*w/v*) sodium azide)
  - from one week to several months at −20 °C, PBS (50% (*w/v*) glycerol, 10 mg/mL BSA, 1 mM DDT, pH 7.4) (HINT 6);
  - for months to years at −80 °C, PBS (50% (*w/v*) glycerol).

### 5.3. Preparation of a Cell System

HeLa cells stably expressing GFP fused to the C-terminus of the NPC scaffold protein POM121 (RRID:CVCL_A9H3) are used for our live cell system and our permeabilized cell system for the single-molecule nuclear transport assays.

#### 5.3.1. Preparation of the Live Cell System

Start a fresh culture of POM121-GFP HeLa cell line from a stock by thawing at 37 °C and inoculating into a 25 cm^2^ culture flask with 5 mL DMEM (Gibco) with 10% (*v/v*) FBS (Gibco) and 1% (*v/v*) penicillin–streptomycin (10,000 U/mL, Gibco). Incubate the cells at 37 °C within a 5% CO_2_ incubator until ~80% confluency is reached. The HeLa cells should be split at least three times before imaging to ensure ideal cellular health and function. 

Twenty-four hours prior to imaging, the HeLa cells should be transferred to a sterile optical dish (MatTek Life Sciences, 35 mm × 50 mm dish, No. 0 coverslip). In addition, the HeLa cells may be grown on a sterile coverslip 48 h in a Petri dish with modified DMEM (10% (*v/v*) FBS and 1% (*v/v*) penicillin–streptomycin (10,000 U/mL)). Then, transfer the coverslip to a glass slide for imaging. For the SPEED microscopy setup, we use a coverslip (Thermo Scientific Gold Seal, No. 0) and glass slide (Thermo Scientific Gold Seal, No. 0). The No. 0 coverslip/slide is used here because of the limited working distance of the objective.

Once the HeLa cells have reached a confluency between 70% and 80%, incubate the Alexa Fluor 647-labeled rAAV2 with the HeLa cells for 15 min at 4 °C with a virus–cell ratio of 10,000:1. 

Incubate the dish at 37 °C in a 5% CO_2_ incubator for 1–3 h. 

After incubation, wash the cells to remove any free AAV particles with 37 °C warmed PBS and add 1–2 mL of 37 °C warmed transport buffer (20 mM HEPES, 110 mM KOAc, 5 mM NaOAc, 2 mM MgOAc, 1 mM EGTA, pH 7.3).

#### 5.3.2. Preparation of the Permeabilized Cell System

Refer to Steps 1 and 2 of Section 5.3.1 “Preparation of the Live Cell System”.

Wash the HeLa cells twice with 37 °C warmed transport buffer. 

Add 1 mL of transport buffer with digitonin (40 μg/mL) and incubate the cells for ~2 min. Then, add ~50 µL of transport buffer with 1.5% polyvinylpyrrolidone (PVP; 360 kDa) to stop the permeabilization of the HeLa cell cytoplasmic membrane (HINT 7). The result will leave several 1–10 µm cavities in the cytoplasmic membrane, while the NE will remain intact [80]. To verify the permeabilization process, the permeabilized cells may be incubated with fluorescently labeled 500 kDa Dextran molecules (Invitrogen). If the molecules enter into the nucleus, then the NE has been compromised. 

Wash the HeLa cells with 1.5% PVP transport buffer to remove any excess digitonin. Then, add 1.5% PVP transport buffer to cells for imaging. 

### 5.4. SPEED Microscopy Tracking of Single Alexa Fluor 647-Labeled AAV through a Single GFP-Labeled NPC in Live and Digitonin-Permeabilized Cells

SPEED microscopy was used to conduct high-speed single molecule tracking of single Alexa Fluor 647-labeled rAAV2 nuclear translocation through the NPC in live and permeabilized POM121-GFP HeLa cells [33]. 

#### 5.4.1. Tracking of Single AAV Particles in Live Cells 

**Figure 4:**
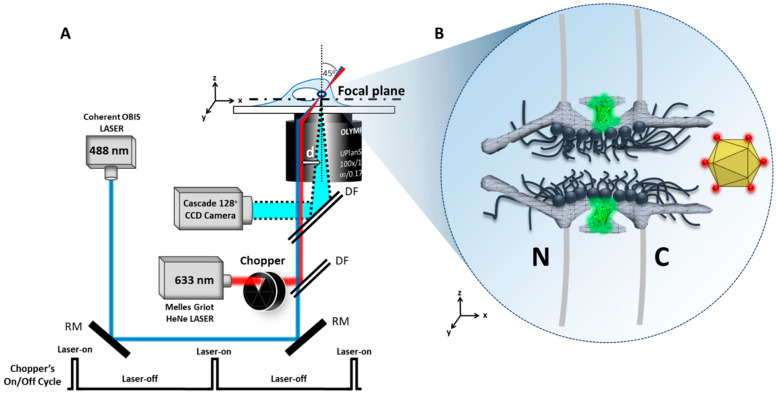
Optical schematic of the SPEED microscope setup. (**A**) Co-alignment of 488 and 633 nm laser beams that were shifted by ~237 µm **d** from the optical axis of the objective to generate an inclined illumination volume at an angle of 45° to the perpendicular direction by using a micrometer stage. The 633 nm laser was chopped by an optical chopper to achieve an on–off laser mode ratio of 1:10 (on–off). The longer laser-off time gives particles transiting the NPC sufficient time to escape from the illumination volume and for fresh fluorescent cargo to diffuse from the cytoplasm or the nucleus into the NPC. (**B**) A single Alexa Fluor 647-labeled AAV2 particle approaching a single GFP-labeled NPC from the cytoplasm **C** to transport to the nucleus **N**.

As shown in Figure 4, equivalent to the illumination pattern of a 488 nm laser, a 633 nm laser is shifted about 237 µm (d) by a micrometer stage off the center of the objective to generate an inclined illumination point spread function at 45° to the perpendicular direction. To reduce photobleaching and phototoxic effects, an optical chopper was placed in the path of the 633 nm laser, creating an on–off laser mode (HINT 8). The 488 nm and 633 nm lasers were used to excite POM121-GFP NPCs and the Alexa Fluor 647 rAAV2, respectively.

Before imaging, the infected cells must incubate for at least 30 min in transport buffer. 

Focus the microscope to the equator of the GFP-fused NE. Target one GFP-labeled NPC at the edge of the NE. Take a 1–2 s image with the 12 µm illumination pattern for an image of the NE.

Switch to the 1 µm illumination pattern and take a 1–2 s image with the 488 nm laser at a 10 µW laser power for the single NPC image. The fluorescent spot of the NPC can then be fitted to a 2D elliptical Gaussian function to determine the centroid position of the NPC (HINT 9). This is further explained in Section 5.6.

If a large number of labeled AAV molecules are within the illumination area, one may need to photobleach the area surrounding the single NPC. With the 633 nm laser at a laser power of 6 mW, illuminate the sample for 60 s or until only background noise is observed. This will improve the signal-to-noise ratio (SNR) for single-molecule tracking.

Set the 633-nm laser to a laser power between 2 and 5 mW and the chopper at 2 Hz to generate a 1:10 on–off ratio. 

In the Slidebook program, respectively set the intensification, gain, and exposure time of the CCD to ~4000, 3, and 2 ms. At a frame rate of 2 ms, 500 frames will be collected per second. For an individual NPC, up to 2 min of videos can be taken of the single-AAV import events. After 2 min, the cell may experience phototoxic effects and the NE may have shifted from the original position (HINT 10).

After single-molecule videos have been taken, switch back to the 12 µm illumination pattern and 488 nm laser and take another NE image. This can be compared with the NE image taken in Step 3 to measure any shift in the NE location (HINT 11).

**Figure 5:**
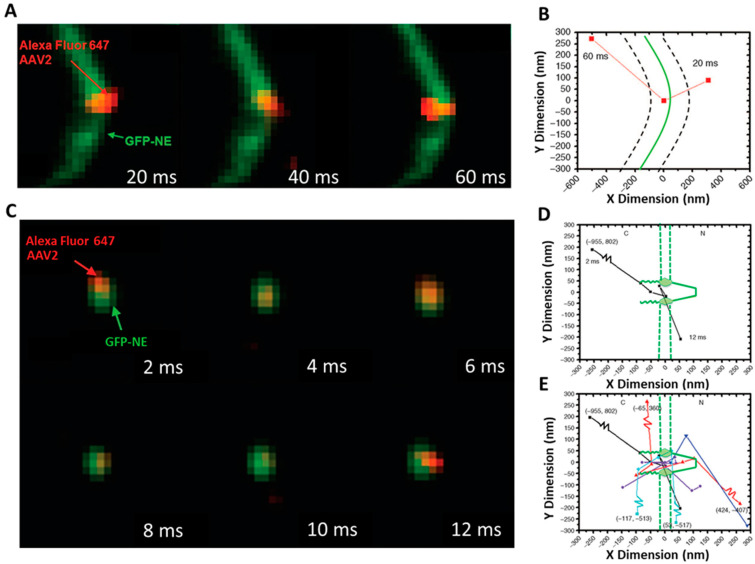
Single-molecule trajectories and 2D spatial locations of AVV2 on the NE and in a single NPC [33]. (**A**) A typical successful single-AVV2 import event captured by wide-field microscopy with a 12 µm illumination area. A single Alexa Fluor 647-labeled AAV2 particle (red spot) started from the cytoplasm, interacted with the GFP-fused NE (green line), and arrived in the nucleus. (**B**) Single-particle tracks (red squares) were acquired by 2D Gaussian fitting to point spread functions in a series of images. The green curve shows the determined position of the middle plane of the NE. The dotted lines indicate −100 and +100 nm from the middle plane of the NE. (**C**) A typical successful single-AAV2 import event captured by SPEED microscopy. A single Alexa Fluor 647-labeled AAV2 particle (red spot) started from the cytoplasm, interacted with a single GFP-fused NPC (green spot), and arrived in the nucleus. (**D**) Single-particle tracks (black squares) and the location of the NPC were acquired by 2D Gaussian fitting to point spread functions in a series of images. (**E**) Compilation of several trajectories (shown in different colors) representing AAV2 particles successfully importing to the nucleus from the cytoplasm through single NPCs. C, cytoplasmic side of the NPC; N, nucleoplasmic side of the NPC. Figures were adapted from our previous publications with permission [33].

An example of a typical trajectory is seen in Figure 5. The fluorescent spot was fitted to a 2D symmetrical Gaussian function and determined the positions of the single-molecule events with respect to the GFP-labeled NPC. Combine all collected trajectories and generate the 2D localization distributions via histograms (Figure 5E). Repeat Steps 3–8 for a different NPC in another cell until a suitable number of locations are obtained (HINT 12). 

#### 5.4.2. Tracking of Single AAV Particles in Permeabilized Cells

Add purified Impβ1 to the permeabilized HeLa cells to reach a final concentration of 0.5–1 µM in 1.5% PVP transport buffer and incubate for 30 min. 

Add 0.1–1 nM of Alexa Fluor 647-rAAV2 to transport buffer near the illumination area. This concentration can be increased further, but if the illumination area becomes oversaturated with signal from the labeled AAV molecules, then the optical dish will need to be rinsed with 1.5% PVP transport buffer or photobleached similarly to Step 5 of Section 5.4.1 “Tracking of Single AAV Particles in Live Cells”. 

Refer to Steps 3 and 4 of Section 5.4.1 “Tracking of Single AAV Particles in Live Cells”.

Refer to Steps 6 through 9 of Section 5.4.1 “Tracking of Single AAV Particles in Live Cells”.

### 5.5. Determining the Copy Number of POM121-GFP and the Labeling Efficiency of the Fluorescently Labeled AAV Particles

Mount a glass slide containing 10 pM concentration of purified GFP in transport buffer on the microscope stage. Give the GFP molecules 30 min to become immobile from non-specific absorption.

Image the single GFP and attain the intensity of single GFP with the 2D Gaussian function.

**Figure 6:**
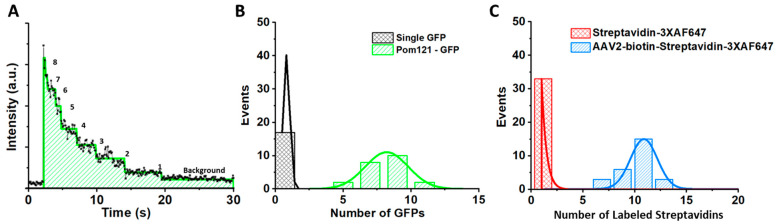
Copy number of GFP-fused NPC and labeling efficiency of Alexa Fluor 647–AAV2 molecules. (**A**) Photobleaching curve of a single GFP-labeled NPC [61]. The steps were determined by the maximum likelihood ratio method. The intensity of single GFP was determined by averaging the step intensity of GFP-POM121 in the NPC of live cells. (**B**) A comparison of the fluorescence intensities from single GFPs to ~8 copies of POM121-GFP found in each NPC [61]. (**C**) A comparison of the fluorescence intensities from single streptavidins with three bounded Alexa Fluor 647 dyes to ~12 copies of 3X Alexa Fluor 647-labeled streptavidin bound to single biotin-labeled AAV2 particles [33].

Replace the glass slide with the POM121-GFP HeLa cells in transport buffer. Then, using the 488 nm laser at a laser power between 0.1 and 0.5 mW and an exposure time between 20 and 50 ms, image a single NPC. Then, one can plot the intensities over the time of the video to generate a photobleaching curve (Figure 6A). Our comparisons between the intensities of single GFP and the POM121-GFP NPC revealed that there were ~8 copies of GFP in each NPC (Figure 6B).

Repeat Steps 1 and 2 with free Alexa Fluor 647 dye in transport buffer. 

Repeat Steps 1 and 2 with the Alexa Fluor 647 streptavidin conjugate. Verify that three of the four binding sites for a single streptavidin are occupied by the Alexa Fluor 647 dye. 

Repeat Steps 1 and 2 for a single biotin–AAV molecule with the Alexa Fluor 647 streptavidin conjugate. Our comparisons between the intensities for single Alexa Fluor 647 streptavidin conjugates and single biotin–AAV molecules with the Alexa Fluor 647 streptavidin conjugates revealed that there were 36 ± 6 copies of Alexa Fluor 647 on each biotin–rAAV2 particle (Figure 6C).

### 5.6. Localization of the NE and a Single NPC on the NE

To visualize the GFP-fused NE, illuminate the entire cell with wide-field epi-fluorescence or a 12 µm lens with the 488 nm laser (Figure 2A,B). If using the 12 µm lens and 488 nm laser, set the laser power to 2–3 mW, exposure time to 20 ms, and the intensification and gain to 4000 and 3, respectively. 

To localize the NE, convert and export the image using the Slidebook Reader software. Then, plot the intensity profile of the x-axis using the open-source ImageJ software. 

Fit the pixel intensities with a Gaussian distribution. The peak position of the Gaussian distribution is the center of the NE for that row. Then, repeat for the remaining rows of the NE image. POM121 is an NPC scaffold protein located in the central region of the NPC and will indicate the center of the NE (Figure 5B). 

The peak positions of a series of such Gaussians are then fit with a second-degree polynomial, yielding the location of the NE within the entire image (Figure 2F).

To visualize a single GFP-fused NPC, illuminate the 1 µm area with the 488 nm laser (Figure 2A,B). Set the laser power to 10 µW, exposure time to 1–2 s, and the intensification and gain to 4000 and 3, respectively (HINT 13 and HINT 14). 

To localize the NPC, convert and export the image using the Slidebook Reader software. Then, use the “Peak Fit” module from the GDSC SMLM plugin found in the open-source ImageJ Fiji software [81]. Additionally, the Glimpse software can be used to fit the single NPC location through a 2D Gaussian function in both the x and y directions (Figure 2G) [68].

### 5.7. Localization Precision of Isolated Fluorescent Spots

The localization precision for fluorescent NPCs, as well as immobile and moving fluorescent Alexa Fluor 647–rAAV2 molecules, was defined as how precisely the central point of each detected fluorescent diffraction-limited spot was determined. For fluorescent NPCs and immobile Alexa Fluor 647–AAV2 molecules, the fluorescent spot was fitted with a 2D elliptical and symmetrical Gaussian, respectively, and the localization precision was determined by the standard deviation (s.d.) of multiple measurements of the central point. For moving Alexa Fluor 647–AAV2 molecules, the fluorescent spot was fitted with a 2D elliptical Gaussian function, and the localization precision (σ) was determined as Equation (1):

(1) $$\sigma =\sqrt{F(16\left(s^{2}+a^{2}\right)/9N+8\pi b^{2}{\left(s^{2}+\frac{a^{2}}{12}\right)}^{2}/a^{2}N^{2}},$$

where *F* is equal to 2, *N* is the number of collected photons, *a* is the effective pixel size of the detector, *b* is the s.d. of the background in photons per pixel, and Equation (2)

(2) $$s=\sqrt{s_{0}^{2}+1/3D\Delta t},$$

where *s*_0_ is the standard deviation of the point spread function in the focal plane, *D* is the diffusion coefficient of substrate in the NPC, and Δ*t* is the image acquisition time [82,83].

Typically, about 3000 signal photons were collected from individual rAAV2 molecules with about 36 Alexa Fluor 647 dyes at a detection frame rate of 2 ms in living cells. The localization precision was 6–9 nm for moving Alexa Fluor 647–rAAV2 molecules. Because of the inevitable vibration of NPCs in the NE of living cells, the localization precision of the NPC centroid was ~6 nm within our 2 min detection time for one single-molecule experiment. The system error of aligned red and green fluorescence channels was determined to be 3.0 ± 0.1 nm. This was determined by the detection of 230 immobile Alexa Fluor 647-labeled GFP molecules on a coverslip. Therefore, the overall tracking precision for Alexa Fluor 647–rAAV2 import through the GFP-labeled NPC in living cells was estimated to be ~9–12 nm. For a further explanation of single-molecule localization fittings, we recommend the 2017 review by A. Diezmann et al. [84]. 

### 5.8. Calculation of Transport Time and Transport Efficiency

After identifying the single-molecule localizations, use the “Trace Diffusion” module from the GDSC SMLM plugin found in the ImageJ Fiji software to find the transport trajectories for a single video [81]. 

Overlay all the trajectories of Alexa Fluor 647–rAAV2 molecules and the GFP-fused NE to identify the locations of all functional NPCs within the imaging area.

From consecutive frames, identify which single-molecule trajectories interact with a single NPC. There are two types of trajectories for molecules that approach within 100 nm of the NE from the cytoplasm. In an abortive event, an AAV molecule interacts with the NPC and returns to the cytoplasm, and in a successful event, an AAV molecule interacts with an NPC and imports into the nucleus. For an abortive event to occur, the first and last points must be >100 nm away from the center of the NE and at least one frame must show a localization within the 100 to 100 nm (cytoplasm to nucleoplasm) axial range of the NPC. This type of trajectory identifies those molecules that did not successfully cross the NE or NPC. A successful event will occur when an AAV molecule’s first and last points are >100 and >100 nm away from the center of the NE, with the first point located in the cytoplasm and the last in the nucleoplasm, with at least one frame showing a localization within the 100 to 100 nm (cytoplasm to nucleoplasm) axial range of the NPC. This type of trajectory identifies that molecules did successfully cross the NE into the nucleus (Figure 5). 

The transport efficiency (*p*) can be calculated by taking the number of successful events and dividing it by total number of successful and abortive events (*n*). Then, from the expected value (p·n), calculate the standard deviation Equation (3):

(3) $$s.d.\;=\sqrt{p\cdot \left(1-p\right)/n}$$

The transport times can be calculated by measuring the number of frames in which a single AAV molecule interacts with the NPC (100 to 100 nm, cytoplasm to nucleoplasm), then multiplying this number by the exposure time. With an exposure time of 2 ms/frame, we multiplied the Alexa Fluor 647–rAAV2 events by 2. 

Put the transport times in a histogram and fit an exponential decay function. The mean life time (τ) will be the average transport time for an AAV molecule interacting with a single NPC. 

## 6. Hints

One may experience a low yield when lysing the HEK293A cells. If this occurs, then increase the temperature of the water bath for the freeze/thaw to 55 °C and aspirate the mixture 3–4 times after the first freeze/thaw with a 23-gauge needle to avoid adding bubbles to the mixture.

To improve the viral yield during the purification step, one may vary the PEG8000 and salt concentrations. Optimizing these concentrations can improve purity and lead to higher infection efficiency. This is discussed in great detail by Guo et al. [85]. 

To reduce expression of potential contamination on the ampicillin agar plates, we recommend that incubation should not exceed 16 h at 37 °C. After 16 h, bacteria with a low-expressing target vector or without the desired vector may express, resulting in low target protein yield. 

IPTG is a lactose metabolite that triggers transcription of the lac operon, where the lacZ gene is replaced with the gene of interest and IPTG is then used to induce gene expression. IPTG is used as a promoter for sequences with the T7 promoter region.

If a low yield from the protein purification is noticed, then decrease the pH and remove the Imidazole from the binding buffer. Then, taking the supernatant from the Ni-NTA pellet step, incubate for 1 h in the new binding buffer at 4 °C while shaking in a covered container. This may increase off-target binding, so additional purification steps may be needed to reach a purity of >90% for single-molecule experiments.

Flash freeze the sample in liquid nitrogen and in small aliquots to reduce protein degradation from a slow freezing process and from multiple freeze–thaw cycles, respectively.

Permeabilization of the cytoplasmic membrane can be monitored in real time by light microscopy. The contrast between the cytoplasm and nucleus will increase as cytoplasmic components flow out of the cell into the extracellular matrix. Similarly, the SNR will increase for the fluorescently labeled NE. 

The precision and photobleaching time of a single AAV2 molecule should be determined before single-molecule experiments. Plate 10 pM of AAV2 molecules on a glass coverslip and wait ~30 min for the molecules to be immobile. After capturing several videos using the same imaging parameters as single-molecule experiments, the intensity and time course of the measured fluorescence can be plotted. The intensity can then be transformed to determine the number of emitted photons to determine the precision using the localization precision formula described in Section 5.7. 

To ensure that an illuminated NPC is perpendicular to the NE, create a histogram of the Gaussian width of the x-dimension to the Gaussian width of the y-dimension from a single GFP–NPC spot. For other cylindrical biological samples, the x and y dimensions would be the short and long axes, respectively. The ratio between the two widths needs to fall between 1.74 and 1.82. Within this range, an illuminated NPC only has a free angle of 1.4° to the direction perpendicular to the NE.

Capture a wide-field image of the NE before and after experiments. This will identify cellular damage or drift of the optical system during imaging. Additionally, in case the high-power laser beam causes some damage to chromatins near the NE, we recommend conducting single-molecule nuclear transport experiments separately from other viral nuclear experiments. 

The NE area around the single-pore images has likely been photobleached from the single GFP–NPC image. Align the wide-field NE images taken before and after single-molecule experiments and recheck the position of the NPC. If the NPC is not localized to the NE, and/or the before and after NE wide-field images do not align, it is likely that the collected data have a bias to the cytoplasm or the nucleoplasm.

The collected single-molecule locations need to be filtered to have good localization precision. For our SPT experiments, we typically use an initial range of <30 nm localization precision before further analyzing the single-molecule data. 

About eight copies of POM121-GFP for individual GFP–NPCs can be identified by obtaining approximately eight-fold fluorescence intensity compared to a single GFP, which also allows for a long exposure time at a low laser power. For the reader’s experiments, we suggest conducting several controls to find the optimal exposure time and laser power to avoid a premature photobleaching of the GFP-NPC.

To simply data analysis, we suggest choosing a GFP–NPC on the equator of the nucleus, so the location of an NPC will be parallel to the y direction of the Cartesian coordinates (x, y) in the CCD camera and perpendicular to the NE.

## Figures and Tables

**Table 1:** Studies of virus–NPC interactions using a variety of fluorescence microscopy techniques. Included here is a non-exhaustive list of fluorescence microscopy techniques applied in the studies of nuclear transport of viral particles.

Virus	Microscopy Technique	Main Observations/Conclusions	References
HSV	Confocal Laser Scanning Microscopy	In permeabilized fixed cells, the interaction between HSV-1 capsid protein pUL25 and Nup214 was identified as an important step for viral genome delivery into the nucleus.	[19]
Adenovirus	Fluorescence Resonance Energy Transfer (FRET) Microscopy	In live cells, the disassembly of adenovirus 5 viral capsids was quantified by a progressive decrease in FRET signal after the capsid docked to the NPC. Two major decreases occurred, suggesting a docked capsid half-life of 3 and 60 min.	[50]
Influenza	Epi-Fluorescence and Single-Particle Tracking	In live cells, the time trajectories of single vRNPs revealed dissociation rate constants ranging from 0.01 to 1 s^−1^ for binding between the vRNPs and the nuclear envelope (NE). In addition, a significant reduction in vRNP nuclear localization was observed in the presence of anti-NPC or wheat germ agglutinin (WGA), suggesting the NPC to be the primary transport route of vRNPs.	[51]
HIV	Super-Resolution Stimulated Emission Depletion (STED) Microscopy	In live cells, the HIV-1 nucleocapsid has been shown to dock at the NE, then import the PIC into the nucleus.	[52]
HBV	Confocal Laser Scanning Microscopy	In permeabilized cells, the transport of intact HBV capsids into the nucleus is facilitated by Impα and Impβ1.	[53]
AAV	SPEED Microscopy	In live cells, single intact AAV2 capsids were found to transport through single NPCs into the nucleus with a nuclear import success rate of ~17%.	[33]

## Data Availability

Data is available upon requests.

## References

[1] Cohen S., Au S., Panté N. (2011). How viruses access the nucleus. Biochim. Biophys. Acta (BBA) Bioenerg..

[2] Rout M.P., Blobel G. (1993). Isolation of the yeast nuclear pore complex. J. Cell Biol..

[3] Görlich D., Kutay U. (1999). Transport between the cell nucleus and the cytoplasm. Annu. Rev. Cell Dev. Biol..

[4] Macara I.G. (2001). Transport into and out of the nucleus. Microbiol. Mol. Biol. Rev..

[5] Yang W. (2013). Distinct, but not completely separate spatial transport routes in the nuclear pore complex. Nucleus.

[6] Maimon T., Elad N., Dahan I., Medalia O. (2012). The human nuclear pore complex as revealed by cryo-electron tomography. Structure.

[7] Ma J., Kelich J.M., Junod S.L., Yang W. (2017). Super-resolution mapping of scaffold nucleoporins in the nuclear pore complex. J. Cell Sci..

[8] Wente S.R., Rout M.P. (2010). The nuclear pore complex and nuclear transport. Cold Spring Harb. Perspect. Biol..

[9] Alber F., Dokudovskaya S., Veenhoff L.M., Zhang W., Kipper J., Devos D., Suprapto A., Karni-Schmidt O., Williams R., Chait B.T. (2007). The molecular architecture of the nuclear pore complex. Nat. Cell Biol..

[10] Vasu S.K., Forbes D.J. (2001). Nuclear pores and nuclear assembly. Curr. Opin. Cell Biol..

[11] Mattaj I.W., Englmeier L. (1998). Nucleocytoplasmic transport: The soluble phase. Annu. Rev. Biochem..

[12] Ma J., Goryaynov A., Yang W. (2016). Super-resolution 3D tomography of interactions and competition in the nuclear pore complex. Nat. Struct. Mol. Biol..

[13] Ma J., Goryaynov A., Sarma A., Yang W. (2012). Self-regulated viscous channel in the nuclear pore complex. Proc. Natl. Acad. Sci. USA.

[14] Lim R.Y.H., Huang N.-P., Köser J., Deng J., Lau K.H.A., Schwarz-Herion K., Fahrenkrog B., Aebi U. (2006). Flexible phenylalanine-glycine nucleoporins as entropic barriers to nucleocytoplasmic transport. Proc. Natl. Acad. Sci. USA.

[15] Zhou Z.H., Dougherty M., Jakana J., He J., Rixon F.J., Chiu W. (2000). Seeing the herpesvirus capsid at 8.5 A. Science.

[16] Ojala P.M., Sodeik B., Ebersold M.W., Kutay U., Helenius A. (2000). Herpes simplex virus type 1 entry into host cells: Reconstitution of capsid binding and uncoating at the nuclear pore complex in vitro. Mol. Cell. Biol..

[17] Sodeik B., Ebersold M.W., Helenius A. (1997). Microtubule-mediated transport of incoming herpes simplex virus 1 capsids to the nucleus. J. Cell Biol..

[18] Copeland A.M., Newcomb W.W., Brown J.C. (2008). Herpes simplex virus replication: Roles of viral proteins and nucleoporins in capsid-nucleus attachment. J. Virol..

[19] Pasdeloup D., Blondel D., Isidro A., Rixon F.J. (2009). Herpesvirus capsid association with the nuclear pore complex and viral DNA release involve the nucleoporin CAN/Nup214 and the capsid protein pUL. J. Virol..

[20] Reddy V.S., Natchiar S., Gritton L., Mullen T.-M., Stewart P., Nemerow G.R. (2010). Crystallization and preliminary X-ray diffraction analysis of human adenovirus. Virology.

[21] Hindley C.E., Lawrence F.J., Matthews D.A. (2007). A role for transportin in the nuclear import of adenovirus core proteins and DNA. Traffic.

[22] Noda T., Sagara H., Yen A., Takada A., Kida H., Cheng R.H., Kawaoka Y. (2006). Architecture of ribonucleoprotein complexes in influenza A virus particles. Nature.

[23] Mattei S., Glass B., Hagen W.J.H., Kräusslich H.-G., Briggs J.A.G. (2016). The structure and flexibility of conical HIV-1 capsids determined within intact virions. Science.

[24] Huang S.-N., Millman I., O’Connell A., Aronoff A., Gault H., Blumberg B.S. (1972). Virus-like particles in Australia antigen-associated hepatitis. An immunoelectron microscopic study of human liver. Am. J. Pathol..

[25] Rayaprolu V., Kruse S., Kant R., Venkatakrishnan B., Movahed N., Brooke D., Lins B., Bennett A., Potter T., McKenna R. (2013). Comparative analysis of adeno-associated virus capsid stability and dynamics. J. Virol..

[26] Jenkins Y., McEntee M., Weis K., Greene W.C. (1998). Characterization of HIV-1 Vpr nuclear import: Analysis of signals and pathways. J. Cell Biol..

[27] Haffar O.K., Popov S., Dubrovsky L., Agostini I., Tang H., Pushkarsky T., Nadler S.G., Bukrinsky M. (2000). Two nuclear localization signals in the HIV-1 matrix protein regulate nuclear import of the HIV-1 pre-integration complex. J. Mol. Biol..

[28] Gallay P., Hope T., Chin D., Trono D. (1997). HIV-1 infection of nondividing cells through the recognition of integrase by the importin/karyopherin pathway. Proc. Natl. Acad. Sci. USA.

[29] Popov S., Rexach M., Zybarth G., Reiling N., Lee M., Ratner L., Lane C.M., Moore M.S., Blobel G., Bukrinsky M. (1998). Viral protein R regulates nuclear import of the HIV-1 pre-integration complex. EMBO J..

[30] Ao Z., Huang G., Yao H., Xu Z., LaBine M., Cochrane A.W., Yao X. (2007). Interaction of human immunodeficiency virus type 1 integrase with cellular nuclear import receptor importin 7 and its impact on viral replication. J. Biol. Chem..

[31] Wang P., Palese P., O’Neill R.E. (1997). The NPI-1/NPI-3 (karyopherin alpha) binding site on the influenza a virus nucleoprotein NP is a nonconventional nuclear localization signal. J. Virol..

[32] O’Neill R.E., Jaskunas R., Blobel G., Palese P., Moroianu J. (1995). Nuclear import of influenza virus RNA can be mediated by viral nucleoprotein and transport factors required for protein import. J. Biol. Chem..

[33] Kelich J.M., Ma J., Dong B., Wang Q., Chin M., Magura C.M., Xiao W., Yang W. (2015). Super-resolution imaging of nuclear import of adeno-associated virus in live cells. Mol. Ther. Methods Clin. Dev..

[34] Popa-Wagner R., Sonntag F., Schmidt K., King J., Kleinschmidt J.A. (2012). Nuclear translocation of adeno-associated virus type 2 capsid proteins for virion assembly. J. Gen. Virol..

[35] Akiba T., Nakayama H., Miyazaki Y., Kanno A., Ishii M., Ohori H. (1987). Relationship between the replication of hepatitis B virus and the localization of virus nucleocapsid antigen (HBcAg) in hepatocytes. J. Gen. Virol..

[36] Kann M., Sodeik B., Vlachou A., Gerlich W.H., Helenius A. (1999). phosphorylation-dependent binding of hepatitis B virus core particles to the nuclear pore complex. J. Cell Biol..

[37] Cohen S., Behzad A.R., Carroll J.B., Panté N. (2006). Parvoviral nuclear import: Bypassing the host nuclear-transport machinery. J. Gen. Virol..

[38] Cohen S., Panté N. (2005). Pushing the envelope: Microinjection of Minute virus of mice into Xenopus oocytes causes damage to the nuclear envelope. J. Gen. Virol..

[39] Hell S.W., Wichmann J. (1994). Breaking the diffraction resolution limit by stimulated emission: Stimulated-emission-depletion fluorescence microscopy. Opt. Lett..

[40] Huang B., Bates M., Zhuang X. (2009). Super-resolution fluorescence microscopy. Annu. Rev. Biochem..

[41] Schermelleh L., Heintzmann R., Leonhardt H. (2010). A guide to super-resolution fluorescence microscopy. J. Cell Biol..

[42] Van Oijen A.M., Köhler J., Schmidt J., Muller M., Brakenhoff G.J. (1999). Far-field fluorescence microscopy beyond the diffraction limit. J. Opt. Soc. Am. A.

[43] Klar T.A., Hell S.W. (1999). Subdiffraction resolution in far-field fluorescence microscopy. Opt. Lett..

[44] Klar T.A., Engel E., Hell S.W. (2001). Breaking Abbe’s diffraction resolution limit in fluorescence microscopy with stimulated emission depletion beams of various shapes. Phys. Rev. E.

[45] Mortensen K.I., Churchman L.S., Spudich J.A., Flyvbjerg H. (2010). Optimized localization analysis for single-molecule tracking and super-resolution microscopy. Nat. Methods.

[46] Erni R., Rossell M.D., Kisielowski C., Dahmen U. (2009). Atomic-resolution imaging with a sub-50-pm electron probe. Phys. Rev. Lett..

[47] Milazzo A.-C., Cheng A., Moeller A., Lyumkis D., Jacovetty E., Polukas J., Ellisman M.H., Xuong N.-H., Carragher B., Potter C.S. (2011). Initial evaluation of a direct detection device detector for single particle cryo-electron microscopy. J. Struct. Biol..

[48] Bammes B.E., Rochat R.H., Jakana J., Chen D.H., Chiu W. (2012). Direct electron detection yields cryo-EM reconstructions at resolutions beyond 3/4 Nyquist frequency. J. Struct. Biol..

[49] Featherstone C., Darby M.K., Gerace L. (1988). A monoclonal antibody against the nuclear pore complex inhibits nucleocytoplasmic transport of protein and RNA in vivo. J. Cell Biol..

[50] Martin-Fernandez M.L., Longshaw S.V., Kirby I., Santis G., Tobin M.J., Clarke D.T., Jones G.R. (2004). Adenovirus type-5 entry and disassembly followed in living cells by FRET, fluorescence anisotropy, and FLIM. Biophys. J..

[51] Babcock H., Chen C., Zhuang X. (2004). Using single-particle tracking to study nuclear trafficking of viral genes. Biophys. J..

[52] Francis A.C., Melikyan G.B. (2018). Single HIV-1 imaging reveals progression of infection through CA-dependent steps of docking at the nuclear pore, uncoating, and nuclear transport. Cell Host Microbe.

[53] Rabe B., Vlachou A., Pante N., Helenius A., Kann M. (2003). Nuclear import of hepatitis B virus capsids and release of the viral genome. Proc. Natl. Acad. Sci. USA.

[54] Finlay D.R., Newmeyer D.D., Price T.M., Forbes D.J. (1987). Inhibition of in vitro nuclear transport by a lectin that binds to nuclear pores. J. Cell Biol..

[55] Li B., Kohler J.J. (2014). Glycosylation of the nuclear pore. Traffic.

[56] Wang D., Tai P.W., Gao G. (2019). Adeno-associated virus vector as a platform for gene therapy delivery. Nat. Rev. Drug Discov..

[57] Ma J., Yang W. (2010). Three-dimensional distribution of transient interactions in the nuclear pore complex obtained from single-molecule snapshots. Proc. Natl. Acad. Sci. USA.

[58] Daigle N., Beaudouin J., Hartnell L., Imreh G., Hallberg E., Lippincott-Schwartz J., Ellenberg J. (2001). Nuclear pore complexes form immobile networks and have a very low turnover in live mammalian cells. J. Cell Biol..

[59] Kubitscheck U., Wedekind P., Zeidler O., Grote M., Peters R. (1996). Single nuclear pores visualized by confocal microscopy and image processing. Biophys. J..

[60] Li Y., Junod S.L., Ruba A., Kelich J.M., Yang W. (2019). Nuclear export of mRNA molecules studied by SPEED microscopy. Methods.

[61] Ma J., Liu Z., Michelotti N., Pitchiaya S., Veerapaneni R., Androsavich J.R., Walter N.G., Yang W. (2013). High-resolution three-dimensional mapping of mRNA export through the nuclear pore. Nat. Commun..

[62] Seisenberger G. (2001). Real-time single-molecule imaging of the infection pathway of an adeno-associated virus. Science.

[63] Xiao P.-J., Samulski R.J. (2012). Cytoplasmic trafficking, endosomal escape, and perinuclear accumulation of adeno-associated virus type 2 particles are facilitated by microtubule network. J. Virol..

[64] Ribbeck K., Görlich D. (2002). The permeability barrier of nuclear pore complexes appears to operate via hydrophobic exclusion. EMBO J..

[65] Tu L.-C., Fu G., Zilman A., Musser S.M. (2013). Large cargo transport by nuclear pores: Implications for the spatial organization of FG-nucleoporins. EMBO J..

[66] Junod S.L., Kelich J.M., Ma J., Yang W. (2020). Nucleocytoplasmic transport of intrinsically disordered proteins studied by high-speed super-resolution microscopy. Protein Sci..

[67] Li Y., Tingey M., Ruba A., Yang W. (2021). High-speed super-resolution imaging of rotationally symmetric structures using SPEED microscopy and 2D-to-3D transformation. Nat. Protoc..

[68] Gelles J. (2014). Gelles-Brandeis. https://github.com/gelles-brandeis/Glimpse.

[69] Herbert A. (2014). Single Molecule Light Microscopy ImageJ Plugins. http://www.sussex.ac.uk/gdsc/intranet/microscopy/UserSupport/AnalysisProtocol/imagej/smlm_plugins/.

[70] Atchison R.W., Casto B.C., Hammon W.M. (1965). Adenovirus-associated defective virus particles. Science.

[71] Hoggan M.D., Blacklow N.R., Rowe W.P. (1966). Studies of small DNA viruses found in various adenovirus preparations: Physical, biological, and immunological characteristics. Proc. Natl. Acad. Sci. USA.

[72] Carter B.J. (2004). Adeno-associated virus and the development of adeno-associated virus vectors: A historical perspective. Mol. Ther..

[73] Matsushita T., Elliger S., Elliger C., Podsakoff G., Villarreal L., Kurtzman G.J., Iwaki Y., Colosi P. (1998). Adeno-associated virus vectors can be efficiently produced without helper virus. Gene Ther..

[74] Grimm D., Zhou S., Nakai H., Thomas C.E., Storm T.A., Fuess S., Matsushita T., Allen J., Surosky R., Lochrie M. (2003). Preclinical in vivo evaluation of pseudotyped adeno-associated virus vectors for liver gene therapy. Blood.

[75] Rose J.A., Hoggan M.D., Shatkin A.J. (1966). Nucleic acid from an adeno-associated virus: Chemical and physical studies. Proc. Natl. Acad. Sci. USA.

[76] Strobel B., Miller F.D., Rist W., Lamla T. (2015). Comparative analysis of cesium chloride- and iodixanol-based purification of recombinant adeno-associated viral vectors for preclinical applications. Hum. Gene Ther. Methods.

[77] Beckett D., Kovaleva E., Schatz P.J. (2008). A minimal peptide substrate in biotin holoenzyme synthetase-catalyzed biotinylation. Protein Sci..

[78] Fairhead M., Howarth M. (2015). Site-specific biotinylation of purified proteins using BirA. Methods Mol. Biol..

[79] Alberghina D., Casella S., Giannetto C., Marafioti S., Piccione G. (2013). Effect of storage time and temperature on the total protein concentration and electrophoretic fractions in equine serum. Can. J. Veter. Res..

[80] Bader M.F., Thiersé D., Aunis D., Ahnert-Hilger G., Gratzl M. (1986). Characterization of hormone and protein release from alpha-toxin-permeabilized chromaffin cells in primary culture. J. Biol. Chem..

[81] GDSC Single Molecule Light Microscopy (SMLM) ImageJ Plugins. https://gdsc-smlm.readthedocs.io/en/latest/.

[82] Thompson R.E., Larson D.R., Webb W.W. (2002). Precise nanometer localization analysis for individual fluorescent probes. Biophys. J..

[83] Quan T., Zeng S., Huang Z.-L. (2010). Localization capability and limitation of electron-multiplying charge-coupled, scientific complementary metal-oxide semiconductor, and charge-coupled devices for superresolution imaging. J. Biomed. Opt..

[84] Von Diezmann A., Shechtman Y., Moerner W.E. (2017). Three-dimensional localization of single molecules for super-resolution imaging and single-particle tracking. Chem. Rev..

[85] Guo P., El-Gohary Y., Prasadan K., Shiota C., Xiao X., Wiersch J., Paredes J., Tulachan S., Gittes G. (2012). Rapid and simplified purification of recombinant adeno-associated virus. J. Virol. Methods.

